# Immunohistochemical localization of urokinase-type plasminogen activator, urokinase-type plasminogen activator receptor and α2-antiplasmin in human corneal perforation: a case report

**DOI:** 10.1186/1471-2415-12-60

**Published:** 2012-11-28

**Authors:** Koji Sugioka, Aya Kodama, Koji Yoshida, Kiyotaka Okada, Masahiko Fukuda, Yoshikazu Shimomura

**Affiliations:** 1 Department of Ophthalmology, Kinki University Faculty of Medicine, 377-2 Ohnohigashi, Osakasayama 589-8511, Japan; 2 Department of Biochemistry, Kinki University Faculty of Medicine, 377-2 Ohnohigashi, Osakasayama, 589-8511, Japan; 3 Department of Physiology and Regenerative Medicine, Kinki University Faculty of Medicine, 377-2 Ohnohigashi, Osakasayama, 589-8511, Japan

**Keywords:** u-PA, u-PAR, Corneal wound healing, α2-antiplasmin, Corneal perforation

## Abstract

**Background:**

Corneal ulceration leading to perforation is associated with infectious and non-infectious destructive conditions in the cornea. The fibrinolytic (plasminogen/plasmin) system is considered to contribute to tissue remodeling in the wound healing process and it is believed to play an important role in proteolysis and fibrosis. To determine the localization of urokinase-type plasminogen activator (u-PA), u-PA receptor (u-PAR) and α2-antiplasmin (α2AP) in the tissue of a corneal perforation, we investigated immunohistochemical expressions of u-PA, u-PAR, α2AP, CD68, and α-smooth muscle actin (α-SMA) in a patient with corneal perforation that developed from an ulcer of no clear cause.

**Case presentation:**

The patient was a 77-year-old woman who presented with a perforated corneal ulcer in her right eye. The cause of her corneal ulcer was unknown. Double immunohistochemistry was performed for the combinations of u-PA with u-PAR, CD68 or α-SMA and α2AP with CD68 or α-SMA to detect the localization of u-PA and α2AP. u-PA and u-PAR co-localization was seen in the corneal ulceration area. u-PA was mainly observed in CD68-positive cells and in some α-SMA positive cells. On the other hand, α2AP was not expressed in CD68-positive cells, but was expressed in α-SMA positive cells.

**Conclusion:**

We identified expression of the u-PA/u-PAR complex and α2AP in a patient with a corneal ulcer. These two molecules are believed to play a crucial role in inflammatory cell recruitment, ECM synthesis and degradation during corneal wound healing.

## Background

Inflammatory cells and activated corneal fibroblasts are main contributors to the wound healing process of corneal ulcers. These cells migrate to the wound edge in response to injury, synthesize enzymes that can degrade the extracellular matrix (ECM) proteins, produce ECM, and contribute to tissue remodeling [1].

Various factors cause prolonged degradation of the ECM, which sometimes leads to corneal perforation. In these processes, inflammatory cells such as macrophages and activated corneal fibroblasts persist in the cornea, causing an imbalance between tissue degradation and remodeling, and eventually worsening the pathological condition.

Fibrinolytic enzymes and matrix metalloproteinase are considered to be crucial factors engaged in the regulation of tissue degradation and remodeling [2,3]. Urokinase-type plasminogen activator (u-PA) is a highly restricted serine protease that binds to a specific receptor (u-PAR) resulting in enhanced cellular proteolysis via the degradation of matrix components or activation of plasminogen [4-7], whereas active plasmin is mainly inhibited by α2-antiplasmin (α2AP) [8,9].

In the current investigation, we observed expressions of u-PA, u-PAR, CD68, α-smooth muscle actin (α-SMA) and α2AP using the cornea of a patient with a corneal perforation.

## Case presentation

**Figure 1:**
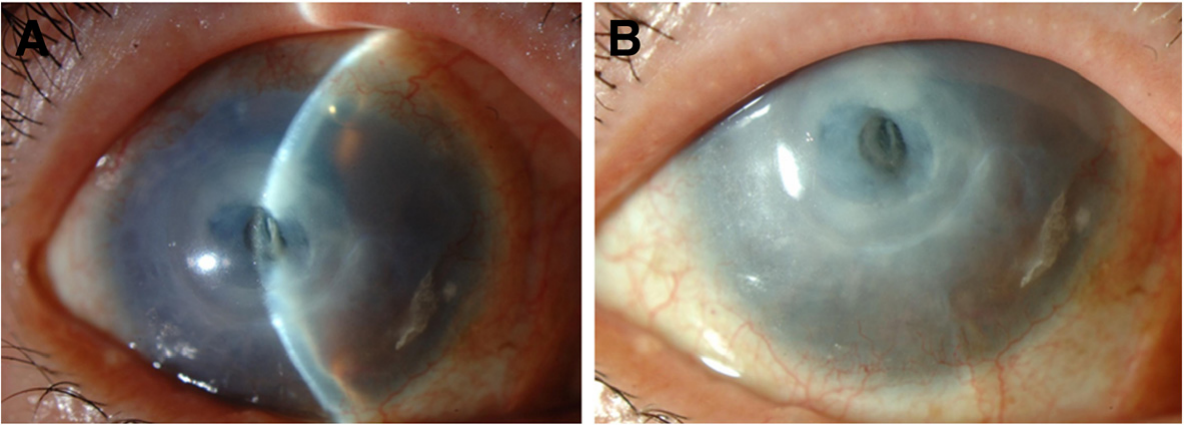
**Clinical findings in the right eye of a 77-year-old female.** The center of the cornea was perforated (**A**) and corneal stromal opacity with fibrosis was seen surrounding the perforation area (**B**).

A 77-year-old woman was referred to the authors in September 2011 for a corneal perforation in her right eye. She complained that she had “hot” tears running out of her eye 2 days before the examination. She exhibited iritis once or twice a year for approximately 15 years. In each episode, she was treated with steroid and antibiotic eye drops in her right eye for a period between 2 weeks and 1 month. On her first visit to our clinic, the center of the cornea was perforated and a corneal stromal opacity with fibrosis was seen surrounding the perforation area (Figure 1). Diagnosis of corneal perforation was made by slit-lamp examination in combination with the Seidel test. The detailed time line of corneal ulcer development was unknown. She had no history of rheumatoid arthritis, dry eyes, eye trauma, corneal surgery or neurotrophic keratopathy. No bacteria or fungi growth was observed in a specimen from the perforated area.

Penetrating keratoplasty was performed in the right eye 2 days after the first visit. The cataract was extracted and an intraocular lens was implanted into the patient’s right eye. A sample of the central corneal button was obtained and processed for histological examination. Our research protocols followed the tenets of the Declaration of Helsinki and the patient gave her informed consent after an explanation of the possible consequences of the study. This study was approved by the IRB/Ethics Committee at Kinki University.

### Light microscopic histological examination and immunohistochemistry

The corneal sample was fixed in Super Fix (Kurabo Industries, Osaka, Japan) and embedded in paraffin. Thin sections (4 μm) were cut and stained with hematoxylin-eosin (HE). Immunostaining for u-PA, u-PAR, CD68 (monocyte and macrophage marker), α-SMA (the most widely observed marker of myofibroblast formation) and α2AP were performed as follows. Sections were deparaffinized and incubated for 20 min at 120°C with Target Retrieval Solution (x10; DAKO, Glostrup, Denmark). They were then cooled down for 30 min at room temperature (RT), washed three times with TNT (0.1 M Tris–HCl, pH7.5, containing 0.15 M NaCl, 0.05% Tween 20) wash buffer, and incubated for 30 min with a mixture of 200 ml methanol and 1 ml H_2_O_2_ at RT. After washing three times with TNT wash buffer, sections were exposed to TNB (0.1 M Tris–HCl, pH7.5, containing 0.15 M NaCl, 0.5% Blocking Reagent [PerkinElmer, Waltham, MA, USA]) blocking buffer for 1 h at RT, and were then incubated with goat anti-u-PA antibody (1:200 dilution in TNB; American Diagnostica, Stamford, CT, USA), rabbit anti-u-PAR antibody (1:200 dilution in TNB; Abcam, Inc, Cambridge, MA, USA), mouse anti-CD68 antibody (1:200 dilution in TNB; Abcam, Inc), rabbit anti- α-SMA antibody (1:200 dilution in TNB; Abcam, Inc), and goat anti-α2AP antibody (1:200 dilution in TNB; Abcam, Inc) at 4°C overnight. After washing three times with TNT wash buffer, sections were incubated for 30 min at RT with an appropriate secondary antibody conjugated with horse-radish peroxidase. Signals were visualized using a tyramide signal amplification system (TSA PLUS; PerkinElmer) according to the manufacturer’s instructions. Sections were photographed under fluorescence microscopy (E800; Canon, Tokyo, Japan) with a CCD camera.

### Double immunohistochemistry

Double immunohistochemistry was performed for the combinations of u-PA with u-PAR, CD68, or α-SMA. Goat anti-u-PA primary antibody was applied and then incubated with anti-goat IgG-HRP, and was revealed using a tyramide signal amplification system labeled with fluorescein isothiocyanate (FITC). Rabbit anti-u-PAR antibody, mouse anti-CD68 antibody or rabbit anti-α-SMA antibody was then applied and was revealed by a tyramide signal amplification system labeled Cyanine 3.

For the combinations of α2AP with CD68 or α-SMA, double immunohistochemical staining was performed. Goat anti-α2AP antibody was applied and then incubated with anti-goat IgG-HRP and was revealed using a tyramide signal amplification system labeled with FITC. Mouse anti-CD68 antibody or rabbit anti-α-SMA antibody was then applied accordingly and was revealed by a tyramide signal amplification system labeled with Cyanine 3 (Cy3). Double-stained sections were examined under a laser scanning confocal microscope (LSM 510 META; Carl Zeiss, Oberkochen, Germany).

## Results

In the current case, corneal perforation was observed in the center of the cornea by slit-lamp examination and a corneal stromal opacity with fibrosis was seen surrounding the perforation area (Figure 1).

**Figure 2:**
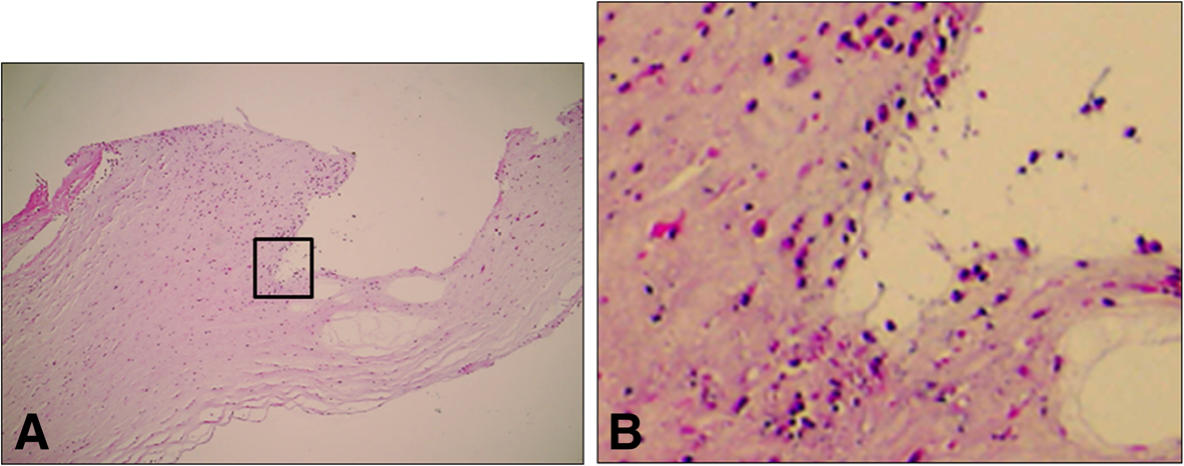
**Photomicrograph of corneal tissue near the perforation.A**, **B**: Hematoxylin & eosin staining, original magnification ×40 and ×400. **B**: Magnified view of the boxed area in **A.**

**Figure 3:**
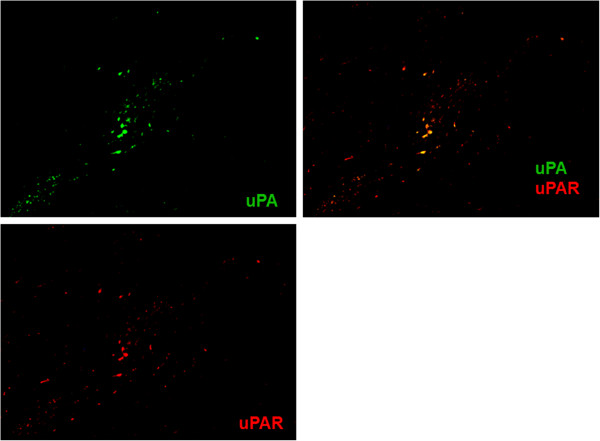
**Double immunostaining for u-PA (green) and u-PAR (red) in corneal tissue near the perforation.** A merged image, with green and red channels shown separately. u-PA and u-PAR co-localization were seen in the corneal ulceration area. Magnification ×100.

**Figure 4:**
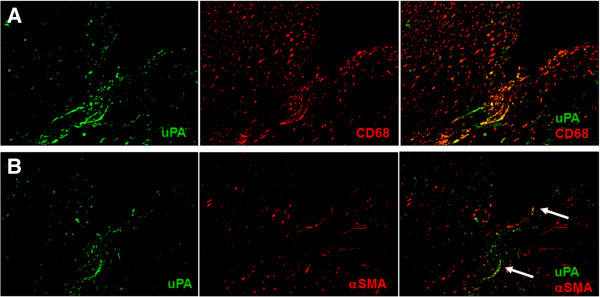
**Expression of u-PA in CD68-positive cells (A) and α-SMA-positive corneal fibroblasts.** (**B**) Double immunostaining for u-PA (green) and CD68 (red) in corneal tissue near the perforation (A). The arrows indicate that some u-PA-positive cells (green) were also positive for α-SMA (red; B).

**Figure 5:**
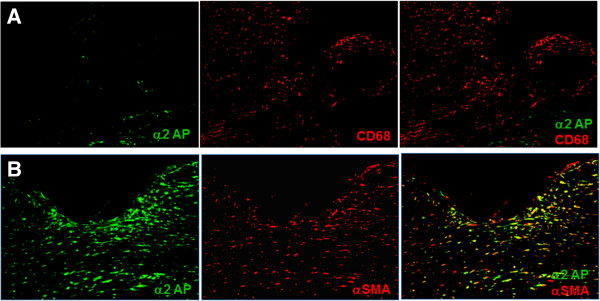
**Expression of α2AP in corneal tissue.** No α2AP expression by CD68-positive cells in corneal tissue near the perforation (**A**). In contrast, α2AP was expressed in α-SMA-positive cells in the corneal stromal opacity area surrounding the perforation (**B**).

HE staining of the corneal section of the current case revealed that both neutrophils and corneal fibroblasts migrated in the stroma near the corneal ulcer (Figure 2). Double immunofluorescence was used to detect expressions of u-PA and u-PAR. As shown in Figure 3, u-PA and u-PAR co-localization was seen in the corneal ulceration area. Furthermore, double immunostaining results of u-PA and CD68 or α-SMA showed cells expressing u-PA were mainly observed in CD68-positive cells (Figure 4A). Some α-SMA positive cells were also positive for u-PA (Figure 4B). On the other hand, α2AP was not expressed in CD68-positive cells (Figure 5A), but was expressed in α-SMA positive cells (Figure 5B).

## Conclusions

The proteolytic activity of plasmin is considered to contribute to tissue remodeling in the wound healing process [10]. Activation of plasminogen by a plasminogen activator is essential in the initiation of fibrinolysis, and there are two types of plasminogen activators; i.e., tissue-type plasminogen activator (t-PA) and u-PA.

u-PA is expressed at the leading edge of the migrating corneal epithelium, whereas t-PA is expressed at a low level during corneal epithelial wound healing [11].

In our case, a large number of migrating inflammatory cells and corneal fibroblasts were observed in the wound edge of the ulcer (Figure 2). This probably indicates repeated and prolonged wound healing reactions. Steroid eye drops are known to delay wound healing and are a factor complicating corneal ulceration. In the present case, long-term steroid treatment may increase the risk of opportunistic corneal infection and delay wound healing. As a result, perforation developed in the central cornea.

Immunohistochemical examination revealed the expression of u-PA in concordance with CD68-positive cells (Figure 4A). At the same time, u-PA was also observed to be partially expressed in α-SMA positive cells (Figure 4B). We suggest that u-PA is mainly produced by CD68-positive cells and also by corneal fibroblasts.

In addition, the expression of u-PAR was observed in the same cells in which u-PA was expressed (Figure 3). This clearly indicated that the infiltrating cells were the u-PA/u-PAR complex. u-PAR expression has been demonstrated in many cell types including macrophages and corneal fibroblasts [12,13]; however, the role of u-PA/u-PAR interaction during ocular inflammation is not clearly understood. Berstein et al. demonstrated that non-cleaved u-PAR (D1D2D3) is present in corneal fibroblasts and cleaved u-PAR (D2D3) is increased in myofibroblasts [14]. The antibody used in our study was made for a C-terminal peptide present both on the intact and cleaved form of the receptor. Therefore, we could not differentiate whether the molecules that reacted to the antibody were non-cleaved or cleaved u-PAR (Figure 3). We hypothesize that u-PAR-bearing cells promote cellular infiltration into the ulcer by binding u-PA and by local degradation of the peripheral corneal stroma.

On the other hand, although expression of α2AP in the cornea has been previously reported [15], the function of α2AP in the cornea has not been elucidated. In our case, interestingly, α2AP expression was observed mostly in the corneal stromal opacity area presumably affected with fibrosis and was barely seen in the ulceration area. Double staining results revealed that α2AP was expressed in α-SMA-positive cells and no α2AP expression was observed in CD68-positive cells (Figures 5A and B).

Many reports have suggested that activated corneal fibroblasts produce ECM and are involved in tissue repair and fibrosis [1,16,17]. The double staining results could be explained by at least two mechanisms: (1) α-SMA-positive fibroblasts produce and secrete α2AP, or (2) α-SMA-positive fibroblasts bind to α2AP. The concordance between α2AP and α-SMA expression may indicate that α2AP is somehow functioning with α-SMA-positive fibroblasts in the fibrotic response.

Kanno et al. [18] demonstrated a new action for α2AP using α2AP knock-out mice where α2AP was associated with tissue fibrosis. This may also be the same mechanism in corneal fibrosis.

In the present case, the wound healing process became prolonged because of the repeated passages of corneal melting and remodeling at the ulcerated area, eventually leading to the development of corneal opacity and perforation. Further investigations are necessary to elucidate the role of u-PA, u-PAR and α2AP in corneal ulcers. These molecules are believed to play a crucial role in inflammatory cell recruitment, ECM synthesis and degradation during corneal wound healing.

To our knowledge, we have identified, for the first time, the expression of the u-PA/u-PAR complex and α2AP in a patient with a corneal ulcer.

### Consent

Written informed consent was obtained from the patient for publication of this case report and any accompanying images. A copy of the written consent is available for review by the series editor of this journal.

## Abbreviations

u-PA: Urokinase-type plasminogen activator; u-PAR: Urokinase-type plasminogen activator receptor; α2AP: α2-antiplasmin; α-SMA: α-smooth muscle actin; ECM: Extracellular matrix.

## Competing interests

The authors declare that they have no competing interests.

## Authors’ contributions

KS collected data, carried out the histological work, and drafted the manuscript. AK was involved in collecting data and carried out the histological work. KY and KO participated in interpreting the data and edited the manuscript. MF was the primary ophthalmologist and performed corneal transplants. YS provided clinical information and participated in its design. All authors read and approved the final manuscript.

## Pre-publication history

The pre-publication history for this paper can be accessed here:

http://www.biomedcentral.com/1471-2415/12/60/prepub
